# Early peritumoral edema change improve prediction of pathological complete response in breast cancer: a multiparametric magnetic resonance imaging-based model

**DOI:** 10.3389/fonc.2026.1671985

**Published:** 2026-01-30

**Authors:** Fuqiang Pan, Yalei Wang, Baoqi Zhang, Yuqing Xin, Heshan Han, Yang Zhang

**Affiliations:** 1 Department of Radiology, Fuyang People’s Hospital of Bengbu Medical University, Fuyang, China; 2 Department of Radiology, Fuyang People’s Hospital of Anhui Medical University, Fuyang, China; 3 Department of Radiology, Fuyang Cancer Hospital, Fuyang, China

**Keywords:** breast neoplasms, neoadjuvant chemotherapy, edema, pathological complete response, magnetic resonance imaging

## Abstract

**Objective:**

To evaluate the independent and additive predictive value of peritumoral edema for pathological complete response (pCR) following neoadjuvant chemotherapy (NAC) in patients with breast cancer.

**Materials and methods:**

Breast cancer patients who underwent NAC between February 2018 and December 2024 were retrospectively enrolled. Based on treatment response, patients were categorized into pCR and non-pCR groups. Clinicopathological characteristics, peritumoral edema, and changes in MRI features (pre-NAC and after two cycles of NAC) were compared between groups. Variables showing statistical significance were included in multivariate logistic regression to identify independent predictors. The additional contribution of edema to predictive performance was assessed using the DeLong test.

**Results:**

A total of 230 patients were included, of whom 96 achieved pCR. Multivariate analysis identified early changes in peritumoral edema, percentage change in apparent diffusion coefficient, percentage change in lesion diameter, and molecular subtype as independent predictors of pCR (P < 0.05). The integration of edema changes with conventional MRI features enhanced predictive accuracy (AUC improved from 0.816–0.844 to 0.851–0.880). A comprehensive prediction model incorporating molecular subtype and MRI features achieved the highest AUC values: 0.899 in the training cohort and 0.882 in the validation cohort.

**Conclusion:**

A predictive model incorporating early peritumoral edema changes, apparent diffusion coefficient, lesion diameter, and molecular subtype achieved an AUC of 0.899, indicating robust predictive performance in predicting pCR among breast cancer patients undergoing NAC. This model may facilitate personalized treatment planning and clinical decision-making.

## 1 Introduction

In 2022, breast cancer was responsible for approximately 666,000 deaths globally, ranking fourth among all cancer-related mortalities and first among women (1). Neoadjuvant chemotherapy (NAC) is the standard treatment for locally advanced breast cancer, aiming to reduce tumor burden, downstage disease before surgery, and improve both breast-conserving rates and survival outcomes (2, 3). Achieving a pathological complete response (pCR) following NAC is associated with significantly improved disease-free survival (DFS) and overall survival (OS) (4). Moreover, patients who demonstrate an early clinical response to NAC are more likely to achieve pCR (5). However, the absence of reliable predictors for pCR may lead to inaccurate assessments, resulting in local recurrence or overtreatment. Therefore, accurately identifying patients unlikely to respond to NAC is critical for avoiding ineffective therapies and enabling individualized treatment planning.

Magnetic resonance imaging (MRI) plays a central role in managing patients undergoing NAC, as treatment decisions depend heavily on accurately assessing therapeutic response. Traditionally, response evaluation has focused on changes in tumor morphology. However, tumor-associated peritumoral edema has emerged as a promising imaging biomarker linked to tumor invasiveness, interstitial inflammation, and poor recurrence-free survival (6, 7).

Despite this, most existing studies have concentrated on pre-treatment edema characteristics, while the dynamic evolution of edema during NAC remains underexplored. In particular, whether early regression of edema after two treatment cycles reflects therapeutic efficacy is still unclear. Studies by Sun et al. and Harada et al. have provided useful insights into edema grading and its association with treatment response, but these were largely limited to baseline imaging features or specific molecular subtypes (e.g., luminal tumors), which restricts their broader clinical applicability (8, 9).

To address these gaps, our study to investigate the dynamic changes in peritumoral edema after two cycles of NAC in breast cancer patients. Specifically, we assess how early changes in edema can predict therapeutic outcomes. In addition, we developed a comprehensive prediction model that integrates peritumoral edema dynamics with various clinicopathological factors, aiming to enhance clinical decision-making and treatment personalization. This approach not only improves the precision of treatment response prediction but also expands the clinical applicability of edema evaluation beyond single-time point assessments and specific tumor subtypes.

Furthermore, this study employs a dual-center design, involving data from Fuyang People’s Hospital and Fuyang Cancer Hospital. This design increases the generalizability and external validity of our findings, ensuring that the predictive model is applicable across different clinical settings and reduces potential bias from a single-center study.

## 2 Materials and methods

### 2.1 Participants

This retrospective, dual-center study included patients with breast cancer who underwent neoadjuvant chemotherapy (NAC) at Fuyang People’s Hospital (Institution 1, n = 80) and Fuyang Cancer Hospital (Institution 2, n = 150) between February 2018 and December 2024.

The study was approved by the Ethics Committee of Fuyang People’s Hospital (approval no. [2025]44). Informed consent was waived due to the retrospective design.

Inclusion criteria were as follows: newly diagnosed breast cancer; pre-biopsy MRI performed before fine-needle aspiration; no evidence of distant metastasis on baseline imaging; MRI performed after two NAC cycles; completion of the full NAC course followed by surgery. Exclusion criteria included: prior biopsy or treatment before NAC initiation; incomplete clinicopathological or follow-up data; missing or low-quality MRI; disease progression or newly detected distant metastasis during NAC; concurrent malignancies; or inability to undergo surgery.

### 2.2 MRI protocol

MRI examinations were performed using a Philips Ingenia CX 3.0T scanner at Institution 1 and a Siemens Avanto 1.5T scanner at Institution 2. The imaging protocol included axial T1-weighted imaging (T1WI; TR/TE = 8.6 ms/4.7 ms); axial fat-suppressed T2WI (TR/TE = 5600 ms/56 ms at Center 1 and 5600 ms/60 ms at Center 2); and diffusion-weighted imaging (DWI) using echo-planar imaging (EPI) with frequency-selective fat suppression and parallel acquisition (b-values: 0 and 800 s/mm²). Dynamic contrast-enhanced MRI (DCE-MRI) was conducted using 3D fast low-angle shot (FLASH) sequences with fat-suppressed axial T1WI. Gadolinium-diethylenetriamine pentaacetic acid (Gd-DTPA) was administered intravenously (0.2 mmol/kg) at 2.5 mL/s via the antecubital vein. One pre-contrast and seven consecutive post-contrast scans were acquired without delay.

### 2.3 Clinicopathological data

Clinicopathological Clinical and pathological variables collected included patient age, molecular subtype (Luminal A, Luminal B, HER2-enriched, or triple-negative breast cancer [TNBC]) before NAC, and postoperative histopathological outcomes. NAC response was evaluated using the Miller–Payne grading system, with Grade 5 defined as pathological complete response (pCR) and Grades 1–4 categorized as non-pCR (n-pCR). Patients were stratified accordingly (2).

### 2.4 Image analysis

**Figure 1:**
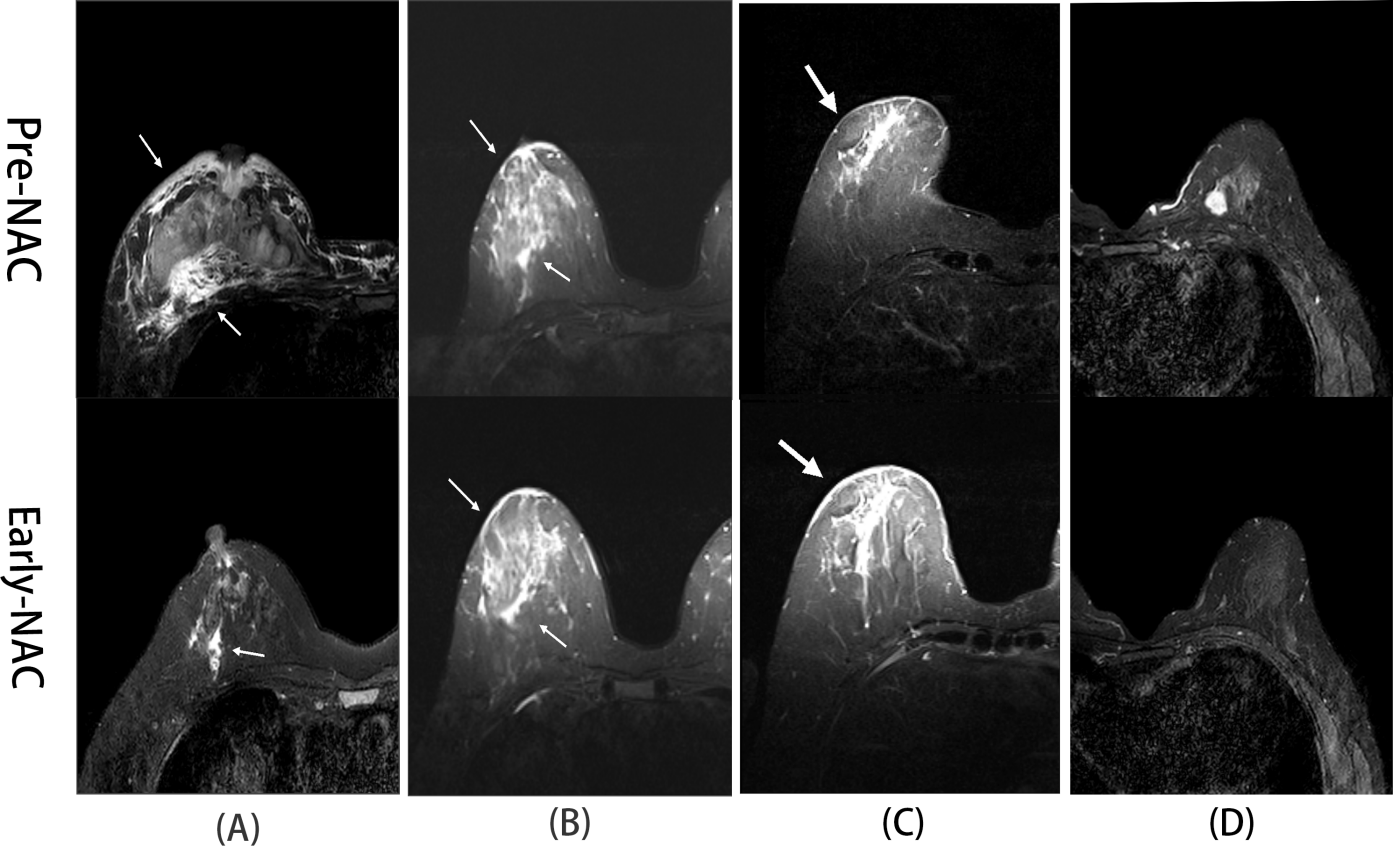
Representative fat-suppressed T2-weighted MR images illustrating peritumoral edema grades (0–3) and their changes during NAC. **(A)** A 46-year-old woman with HER2-positive breast cancer. Pre-NAC T2WI (white arrows) shows diffuse edema (Grade 3) in the right breast. After two NAC cycles (white arrows), peritumoral edema (Grade 1) was observed, indicating decreased edema. Postoperative pathology confirmed a pathological complete response (pCR). **(B)** A 55-year-old woman with HER2-positive breast cancer. Pre-NAC T2WI (white arrows) reveals diffuse edema (Grade 3) in the right breast. After two NAC cycles (white arrows), diffuse edema persists with no significant change, and postoperative pathology confirmed a non-pCR. **(C)** A 62-year-old woman with triple-negative breast cancer (TNBC). Pre-NAC T2WI (white arrows) shows subcutaneous edema (Grade 2) in the right breast. Edema increased in extent (Grade 3) after two NAC cycles (white arrows), and postoperative pathology confirmed a non-pCR. **(D)** A 36-year-old woman with luminal A-type breast cancer. T2WI before and after two NAC cycles showed no detectable edema at either time point (Grade 0), and postoperative pathology confirmed a pathological complete response (pCR).

All MRI data were analyzed using the IntelliSpace Portal Version 7.0 (Philips Healthcare). Baseline MRI characteristics assessed included tumor morphology (regular vs. irregular), enhancement pattern (edge vs. non-edge enhancement), and edema type. Edema was graded by anatomical distribution as follows: Grade 0 (no edema), Grade 1 (peritumoral edema), Grade 2 (prepectoral or subcutaneous edema), and Grade 3 (diffuse edema; **Figure 1**) (8). Quantitative MRI features included maximum lesion diameter (LD) and apparent diffusion coefficient (ADC). Regions exhibiting cystic or liquefactive changes were excluded from ADC measurement to ensure accuracy.

Early-NAC MRI was obtained after two treatment cycles. Edema response was classified into four categories: decreased (reduced grade or extent), stable (no change), increased (elevated grade or extent), or persistently absent. Tumor size and ADC changes during NAC were calculated using the following formulas: ΔLD% = [(LDpre − LDearly)/LDpre] × 100%, and ΔADC% = [(ADCearly − ADCpre)/ADCpre] × 100%, where LDpre and ADCpre are baseline values, and LD early and ADC early refer to values after the second NAC cycle.

Two experienced breast radiologists (with 8 and 10 years of experience, respectively) independently graded peritumoral edema. Discrepancies were resolved by consensus.

### 2.5 Statistical analysis

Statistical analyses were conducted using R software (version 4.4.0) and SPSS (version 27.0). Continuous variables with normal distributions were compared using independent-sample *t*-tests and are reported as mean ± standard deviation (SD). Categorical variables were analyzed using the chi-square (*χ*²) test and are presented as frequencies and percentages. Univariate and multivariate logistic regression analyses were performed to identify independent predictors of pathological complete response (pCR). A P-value of < 0.05 was considered statistically significant. Model performance was assessed using receiver operating characteristic (ROC) curve analysis. The incremental predictive value of early edema changes was evaluated with the DeLong test. Model discrimination, calibration, and clinical utility were assessed using the area under the ROC curve (AUC), calibration plots, and decision curve analysis (DCA), respectively. Model goodness-of-fit was tested using the Hosmer–Lemeshow test. To ensure inter-reader reproducibility, Kappa statistics were calculated to evaluate the agreement between the two radiologists.

## 3 Results

### 3.1 Baseline characteristics of the development and validation cohorts

A total of 230 female patients were included in the study—80 from Institution 1 and 150 from Institution 2. Among them, 96 patients (41.74%) achieved pCR, while 134 (58.26%) did not.

Patients were randomly allocated to a development cohort (n = 161) and a validation cohort (n = 69) using a 7:3 ratio. No significant differences were observed in clinical or pathological characteristics between the two cohorts, indicating good baseline comparability **Table 1**, P > 0.05).

**Table 1:** Baseline characteristics of the development and validation cohorts.

Variable	Total (n = 230)	Validation cohort (n = 69)	Development cohort (n = 161)	*t/χ²*	P-value
pCR				*χ*² = 1.23	0.268
n-pCR	134 (58.26%)	44 (63.77%)	90 (55.90%)		
pCR	96 (41.74%)	29 (36.23%)	71 (44.10%)		
Age (y)	50.33 ± 9.78	50.43 ± 11.22	50.29 ± 9.14	*t* = 0.10	0.919
Molecular subtype				*χ*² = 2.20	0.532
Luminal A	49 (21.30%)	15 (21.74%)	34 (21.12%)		
Luminal B	65 (28.26%)	17 (24.64%)	48 (29.81%)		
HER2+	75 (32.61%)	21 (30.43%)	54 (33.54%)		
TNBC	41 (17.83%)	16 (23.19%)	25 (15.53%)		
Lesion morphology				*χ*² = 0.39	0.532
Regular	70 (30.43%)	19(27.54%)	51 (31.68%)		
Irregular	160 (69.57%)	50 (72.46%)	110 (68.32%)		
Lymphadenopathy				*χ*² = 0.58	0.448
Absent	40 (17.39%)	10 (14.49%)	30 (18.63%)		
Present	190 (82.61%)	59 (85.51%)	131 (81.37%)		
Enhancement pattern				*χ*² = 1.78	0.182
Non-rim enhancement	164 (71.30%)	45 (65.22%)	119 (73.91%)		
Rim enhancement	66 (28.70%)	24 (34.78%)	42 (26.09%)		
Baseline edema grade (pre-NAC)				*χ*² = 0.11	0.991
Grade 0	36 (15.65%)	11 (15.94%)	25 (15.53%)		
Grade 1	107 (46.52%)	33 (47.83%)	74 (45.96%)		
Grade 2	31 (13.48%)	9 (13.04%)	22 (13.66%)		
Grade 3	56 (24.35%)	16 (23.19%)	40 (24.84%)		
ΔEdema_early_				*χ*² = 2.13	0.545
Decreased	96 (41.74%)	25 (36.23%)	71 (44.10%)		
Stable	78 (33.91%)	27 (39.13%)	51 (31.68%)		
Increased	22 (9.57%)	8 (11.59%)	14 (8.70%)		
Always without edema	34 (14.78%)	9 (13.04%)	25 (15.53%)		
ΔLD%	42.26 ± 26.12	39.65 ± 21.95	43.38 ± 27.70	*t* = −1.09	0.279
ΔADC%	33.03 ± 22.16	32.00 ± 21.50	33.48 ± 22.49	*t* = −0.46	0.645

Note: t = t-test; χ² = chi-square test.

“Δ” represents the relative changes in features between pre-NAC and early-NAC stage.

ΔEdema early, early change in peritumoral edema between baseline and after two cycles of neoadjuvant chemotherapy (NAC); ΔLD%, percentage change in lesion diameter; ΔADC%, percentage change in apparent diffusion coefficient.

### 3.2 Logistic analysis of clinical, pathological, and imaging features between the pCR and non-pCR groups

In the development cohort, the pCR and non-pCR groups showed significant differences in early changes in tumor longest diameter (ΔLD%), apparent diffusion coefficient (ΔADC%), molecular subtype, and early edema changes (P < 0.05). These variables were included in the multivariate logistic regression analysis, which identified molecular subtype, ΔADC%, ΔLD%, and early edema change as independent predictors of pCR (P < 0.05; **Table 2**).

**Table 2:** Univariate and multivariate logistic regression analyses of predictors for pCR in the development cohort (n = 161).

Variables	Univariate analysis					Multivariable analysis				
	Total (n = 161)	Non-pCR (n = 90)	pCR (n = 71)	*t/χ²*	P-value	β	S.E	Z	P	OR (95% CI)
Age (y)	50.29 ± 9.14	49.96 ± 9.85	50.72 ± 8.19	*t* = −0.52	0.601					
Molecular subtype				*χ*² = 33.21	<0.001					
Luminal A	34 (21.12%)	30 (33.33%)	4 (5.63%)							1.00 (Reference)
Luminal B	48 (29.81%)	31 (34.44%)	17 (23.94%)			1.54	0.76	2.03	0.042	4.64 (1.06–20.42)
HER2+	54 (33.54%)	15 (16.67%)	39 (54.93%)			2.53	0.78	3.25	0.001	12.52 (2.73–57.40)
TNBC	25 (15.53%)	14 (15.56%)	11 (15.49%)			1.63	0.89	1.84	0.066	5.12 (0.90–29.19)
Lesion morphology				*χ*² = 3.51	0.061					
Regular	51 (31.68%)	34 (37.78%)	17 (23.94%)							
Irregular	110 (68.32%)	56 (62.22%)	54 (76.06%)							
Lymphadenopathy				*χ*² = 0.10	0.754					
Absent	30 (18.63%)	16 (17.78%)	14 (19.72%)							
Present	131 (81.37%)	74 (82.22%)	57 (80.28%)							
Enhancement pattern				*χ*² = 1.62	0.203					
Non-rim enhancement	119 (73.91%)	63 (70.00%)	56 (78.87%)							
Rim enhancement	42 (26.09%)	27 (30.00%)	15 (21.13%)							
Baseline edema grade (pre-NAC)				*χ*² = 2.12	0.547					
Grade 0	25 (15.53%)	12 (13.33%)	13 (18.31%)							
Grade 1	74 (45.96%)	40 (44.44%)	34 (47.89%)							
Grade 2	22 (13.66%)	15 (16.67)	7 (9.86%)							
Grade 3	40 (24.84%)	23 (25.56%)	17 (23.94%)							
ΔEdema_early_				*χ*² = 41.81	<0.001					
Decreased	71 (44.10%)	23 (25.56%)	48 (67.61%)							1.00 (Reference)
Stable	51 (31.68%)	43 (47.78%)	8 (11.27%)			−1.42	0.54	−2.61	0.009	0.24 (0.08–0.70)
Increased	14 (8.70%)	13 (14.44%)	1 (1.41%)			−2.59	1.30	−1.99	0.047	0.08 (0.01−0.97)
Always without edema	25 (15.53%)	11 (12.22%)	14 (19.72%)			0.43	0.68	0.64	0.524	1.54 (0.41–5.82)
ΔLD%	43.38 ± 27.70	29.58 ± 22.12	60.87 ± 24.00	*t* = −8.58	<0.001	0.03	0.01	2.97	0.003	1.03 (1.01−1.05)
ΔADC%	33.48 ± 22.49	24.21 ± 17.41	45.22 ± 22.81	*t* = −6.42	<0.001	0.03	0.01	2.04	0.042	1.03 (1.01–1.06)

t, t-test; χ², chi-square test, β, regression coefficient; CI, confidence interval; SE, standard error; Z, test statistic; OR, odds ratio.

### 3.3 Additional predictive value of edema change for treatment response

To evaluate the added predictive value of early edema change, we compared two models within the development cohort. Model 1 included only conventional MRI parameters (ΔLD% and ΔADC%), whereas Model 2 incorporated early edema change in addition to ΔLD% and ΔADC%. The inclusion of early edema change significantly improved the prediction of pCR (AUC = 0.880, 95% CI = 0.830–0.931 vs. 0.844, 95% CI = 0.784–0.904; P = 0.037, DeLong test; **Table 3**). Further enhancement was achieved with Model 3, which integrated molecular subtype alongside ΔLD%, ΔADC%, and early edema change. This model attained the highest AUC of 0.899 (AUC = 0.899, 95% CI = 0.853–0.946vs. 0.844, 95% CI = 0.785–0.904; P = 0.008; **Table 3**), representing a statistically significant improvement over Model 1.

**Table 3:** Added predictive value of edema change for pCR.

Model	Predictors	AUC (95% CI)	
		Development	Validation
Model 1	ΔLD%+ΔADC%	0.844 (0.784–0.904)	0.816 (0.702–0.929)
Model 2	ΔLD%+ΔADC%+ ΔEdema_early_	0.880 (0.830–0.931)	0.851 (0.747–0.955)
Model 3	ΔLD%+ΔADC%+ ΔEdema_early_ + molecular subtype	0.899 (0.853–0.946)	0.882 (0.785–0.979)

AUC, area under the curve; CI, confidence interval; ΔLD%, percentage change in lesion diameter; ΔADC%, percentage change in apparent diffusion coefficient; ΔEdema early, early change in peritumoral edema between baseline and after two cycles of neoadjuvant chemotherapy (NAC).

### 3.4 Construction and evaluation of the nomogram-based predictive model

**Figure 2:**
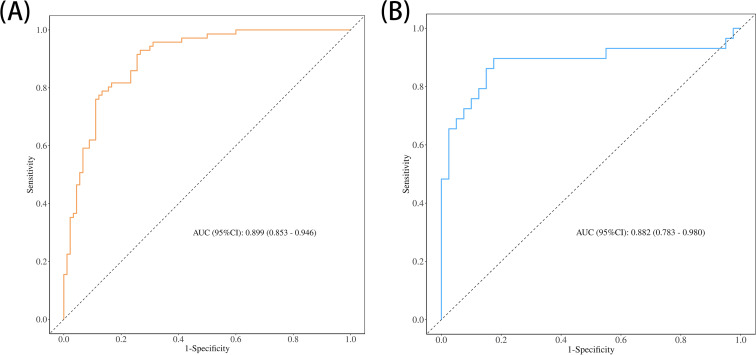
**(A, B)** Receiver operating characteristic (ROC) curves for the development cohort and validation cohort.

**Figure 3:**
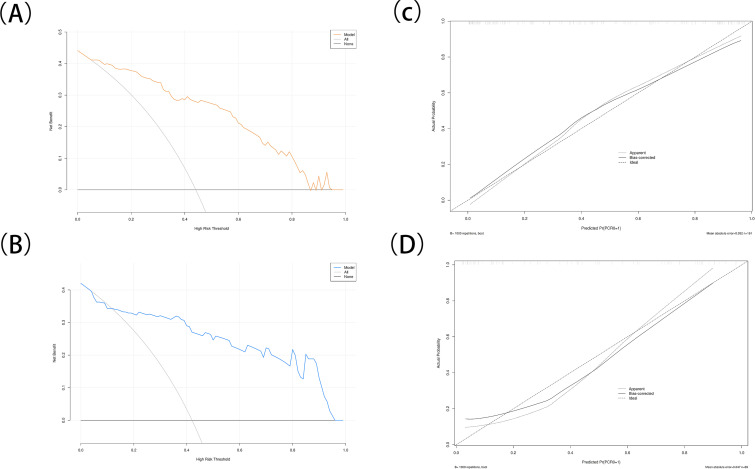
**(A, B)** Decision curve analysis (DCA) for the development cohort and validation cohort. **(C, D)** Calibration curves for the development cohort and validation cohort.

**Figure 4:**
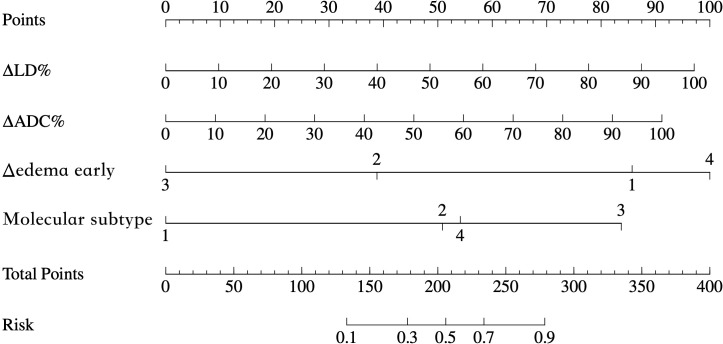
Nomogram integrating multiparametric MRI features and molecular subtype for an individualized prediction of pCR. The nomogram includes ΔADC%, ΔLD%, Δedema change, and molecular subtype. Total points correspond to the estimated probability of achieving pCR after NAC.

A multivariable prediction model was subsequently constructed based on the four independent predictors identified. Receiver operating characteristic (ROC) curves were plotted for both the development and validation cohorts (**Figure 2**). The combined model achieved an AUC of 0.899 in the development cohort and 0.882 in the validation cohort. Sensitivity and specificity were 82.0% and 93.0% in the development cohort, and 65.9% and 80.0% in the validation cohort, respectively. Calibration analysis showed strong concordance between predicted and observed outcomes (Hosmer–Lemeshow test: χ² = 10.426, P = 0.2364). Decision curve analysis (DCA) demonstrated that the combined model offered substantial net clinical benefit across a broad range of threshold probabilities (**Figure 3**), and a corresponding nomogram was developed to visually represent the model (**Figure 4**). The edema grading was performed independently by two radiologists, and the inter-reader reproducibility was assessed using Kappa statistics. The Kappa value was 0.84(P<0.001), indicating a high degree of consistency between the two readers.

## 4 Discussion

This retrospective study included 230 breast cancer patients who received NAC at two institutions. Early changes in peritumoral edema following two cycles of treatment—along with percentage changes in apparent diffusion coefficient (ΔADC%), lesion diameter (ΔLD%), and molecular subtype—were identified as independent predictors of pCR. Notably, early edema change showed strong predictive power (P < 0.001) and offered incremental value beyond conventional MRI metrics such as ΔADC% and ΔLD% (P = 0.035). These findings suggest that edema dynamics may serve as a sensitive marker of therapy-induced alterations in the tumor microenvironment. By integrating multiparametric MRI features with molecular subtype stratification, we constructed a composite model that demonstrated excellent performance, with an AUC of 0.899 in the training cohort and 0.882 in the validation cohort. The improvement in AUC from 0.844 to 0.899 (P = 0.008) underscores the added value of incorporating early changes in peritumoral edema into the predictive model. This statistically significant enhancement demonstrates that early-phase NAC evaluations, which include dynamic changes in edema, offer a more accurate prediction of pathological complete response (pCR). Clinically, this improvement enables more personalized treatment strategies by better identifying patients who are likely to benefit from treatment, while avoiding unnecessary therapies for those less likely to respond. Furthermore, the dual-center design enhances the external validity of our results, making them more applicable in clinical settings.

MRI is widely recognized as a reliable modality for evaluating breast edema (9). In the context of breast cancer, edema has been linked to angiogenesis, increased vascular permeability, peritumoral hyaluronic acid accumulation, lymphovascular invasion, and mechanical compression by the tumor mass (10, 11). A reduction in the extent or grade of edema often signals a favorable response to therapy, as chemotherapeutic agents—particularly taxanes—can suppress inflammation and reduce vascular permeability (12). When combined with anti-angiogenic agents, these therapies promote vascular normalization, decrease immature neovessel formation, and enhance lymphatic drainage through tumor shrinkage and alleviation of mechanical pressure (13). In our study, edema regression was associated with reduction in lesion diameter (ΔLD%), indicating tumor regression, and increased ADC values (ΔADC%), reflecting reduced cellularity. The concurrent presence of edema regression and ADC elevation may therefore serve as an imaging signature of tumor deactivation. Conversely, Persistent or increased edema may indicate suboptimal treatment response or disease progression. This could reflect ongoing tumor activity, persistent inflammation, vascular leakage, insufficient treatment duration, or lymphatic dysfunction (e.g., fibrosis or destruction). Importantly, molecular subtype also influences edema resolution: slow-proliferating luminal tumors may show delayed edema regression, while persistent edema in triple-negative breast cancer (TNBC) often signifies poor therapeutic response or emerging chemoresistance (8).

Compared with the study by Harada et al. (6), which assessed baseline edema grades prior to NAC, our findings highlight that early changes in peritumoral edema (reduction vs. stable/increased) offer greater predictive value than static pre-treatment measurements. Single time-point assessments may underestimate the prognostic significance of edema by failing to capture treatment-induced remodeling of the tumor microenvironment. In contrast, our study shifts the focus to the early phase of NAC, enhancing predictive sensitivity by capturing dynamic edema changes—such as early regression—a strategy aligned with Yao et al. (14). Similarly, Sun et al. (8) reported that early edema reduction correlated with treatment response in luminal-type breast cancer. Unlike prior studies that focused on individual subtypes, our analysis included all four molecular subtypes and found that persistent or increased edema after early NAC was a negative predictor of pCR. Patients exhibiting stable or increased edema after two NAC cycles had significantly lower odds of achieving pCR (OR = 0.24 and 0.08; P = 0.004 and 0.016, respectively). Conversely, early edema regression was strongly associated with favorable pathological response, consistent with Liang et al. (15), who observed that concurrent tumor shrinkage and edema improvement during NAC were linked to better outcomes. Collectively, these results underscore the clinical utility of monitoring early edema dynamics as a noninvasive, early predictor of NAC efficacy. This observation is consistent with the biological processes of vascular normalization, reduced vascular permeability, and improved lymphatic drainage that occur during effective chemotherapy (16).

This study integrates dynamic imaging biomarkers with the biological heterogeneity of molecular subtypes, thereby addressing the limitations of single-parameter models. Early-phase NAC evaluation provides more clinically relevant prognostic information than assessments made at treatment completion. Specifically, ΔADC% reflects chemotherapy-induced reductions in tumor cellularity, in line with findings by Partridge et al. (17), who showed that mid-treatment ADC changes predicted pCR. Likewise, ΔLD% quantifies tumor volume reduction, and both ADC elevation and lesion diameter shrinkage during NAC have been validated as early imaging biomarkers for pCR prediction (18, 19). Stratification by molecular subtype further improved the model’s predictive accuracy. The calibration curve demonstrated strong agreement between predicted and observed probabilities (Hosmer–Lemeshow P = 0.236). Internal validation confirmed robust model performance in the validation cohort, and decision curve analysis showed a high net clinical benefit. The resulting nomogram serves as a practical tool for individualized treatment planning. By dynamically tracking edema and other imaging biomarkers, this model facilitates early identification of low-risk candidates for breast-conserving surgery, potentially reducing overtreatment, while also enabling timely chemotherapy modifications to avoid ineffective regimens. This integrative approach lays a foundation for personalized therapy, especially in predicting treatment response in highly proliferative subtypes such as HER2-positive and triple-negative breast cancers.

In this study, baseline edema grade before NAC was not significantly associated with pCR (P = 0.547), consistent with findings by Chen et al. (20), but contrasting with earlier studies. Bae et al. (21) reported that patients with no edema or only peritumoral edema had higher pCR rates, while those with diffuse edema responded poorly. This discrepancy may reflect inter-subtype variations in edema grade (21–23) or may stem from limited sample size and reduced statistical power in our cohort.

This study has several limitations. First, its retrospective design may introduce selection bias. Second, although data were collected from two centers, external validation in an independent cohort is still needed. Third, minor variations in MRI acquisition protocols between scanners could introduce measurement variability. Finally, the relatively small number of cases per molecular subtype may limit subgroup analyses.

## 5 Conclusions

In summary, this study demonstrates and validates the added predictive value of early edema changes for pCR prediction. By combining early edema dynamics, MRI biomarkers (ΔADC%, ΔLD%), and molecular subtype, we developed a predictive model achieving AUC > 0.85 in both training and validation cohorts—indicating strong clinical utility. The nomogram derived from this model may support clinicians in tailoring individualized treatment strategies and enhancing NAC response prediction.

## Data Availability

The raw data supporting the conclusions of this article will be made available by the authors, without undue reservation.

## References

[1] Bray F Laversanne M Sung H Ferlay J Siegel RL Soerjomataram I . Global cancer statistics 2022: GLOBOCAN estimates of incidence and mortality worldwide for 36 cancers in 185 countries. CA Cancer J Clin. (2024) 74:229–63. doi: 10.3322/caac.21834, PMID: 38572751

[2] Yee D DeMichele AM Yau C Isaacs C Symmans WF Albain KS . Association of event-free and distant recurrence–free survival with individual-level pathologic complete response in neoadjuvant treatment of stages 2 and 3 breast cancer: three-year follow-up analysis for the I-SPY2 adaptively randomized clinical trial. JAMA Oncol. (2020) 6:1355. doi: 10.1001/jamaoncol.2020.2535, PMID: 32701140 PMC7378873

[3] Spring LM Fell G Arfe A Sharma C Greenup R Reynolds KL . Pathologic complete response after neoadjuvant chemotherapy and impact on breast cancer recurrence and survival: a comprehensive meta-analysis. Clin Cancer Res. (2020) 26:2838–48. doi: 10.1158/1078-0432.CCR-19-3492, PMID: 32046998 PMC7299787

[4] Xie L Wang Y Wan A Huang L Wang Q Tang W . Research trends of neoadjuvant therapy for breast cancer: a bibliometric analysis. Hum Vaccin Immunother. (2025) 21:2460272. doi: 10.1080/21645515.2025.2460272, PMID: 39904891 PMC11801352

[5] Von Minckwitz G Loibl S . *In vivo* chemosensitivity adapted preoperative chemotherapy in patients with early stage breast cancer: the Gepartrio pilot study. Ann Oncol. (2005) 16:1560–61. doi: 10.1093/annonc/mdi283, PMID: 15598939

[6] Harada TL Uematsu T Nakashima K Kawabata T Nishimura S Takahashi K . Evaluation of breast edema findings at T2-weighted breast MRI is useful for diagnosing occult inflammatory breast cancer and can predict prognosis after neoadjuvant chemotherapy. Radiology. (2021) 299:53–2. doi: 10.1148/radiol.2021202604, PMID: 33560188

[7] Fowler AM Mankoff DA Joe BN . Imaging neoadjuvant therapy response in breast cancer. Radiology. (2017) 285:358–75. doi: 10.1148/radiol.2017170180, PMID: 29045232

[8] Sun S Zhou J Bai Y Gao W Lin L Jiang T . Role of oedema and shrinkage patterns for prediction of response to neoadjuvant chemotherapy and survival outcomes in luminal breast cancer. Clin Radiol. (2024) 79:e1010–20. doi: 10.1016/j.crad.2024.04.021, PMID: 38830784

[9] Harada TL Uematsu T Nakashima K Sugino T Nishimura S Takahashi K . Is the presence of edema and necrosis on T2WI pretreatment breast MRI the key to predict pCR of triple negative breast cancer? Eur Radiol. (2020) 30:3363–70. doi: 10.1007/s00330-020-06662-7, PMID: 32062698

[10] Baltzer PAT Yang F Dietzel M Herzog A Simon A Vag T . Sensitivity and specificity of unilateral edema on T2w-TSE sequences in MR-mammography considering 974 histologically verified lesions. Breast J. (2010) 16:233–39. doi: 10.1111/j.1524-4741.2010.00915.x, PMID: 20565468

[11] Park NJ-Y Jeong JY Park JY Kim HJ Park CS Lee J . Peritumoral edema in breast cancer at preoperative MRI: an interpretative study with histopathological review toward understanding tumor microenvironment. Sci Rep. (2021) 11:12992. doi: 10.1038/s41598-021-92283-z, PMID: 34155253 PMC8217499

[12] Jain RK . Normalization of tumor vasculature: an emerging concept in antiangiogenic therapy. Science. (2005) 307:58–62. doi: 10.1126/science.1104819, PMID: 15637262

[13] Malhaire C Selhane F Saint-Martin M-J Cockenpot V Akl P Laas E . Exploring the added value of pretherapeutic MR descriptors in predicting breast cancer pathologic complete response to neoadjuvant chemotherapy. Eur Radiol. (2023) 33:8142–54. doi: 10.1007/s00330-023-09797-5, PMID: 37318605

[14] Yao L Liu X Wang M Yu K Xu S Qiu P . Predicting pathological complete response in breast cancer after two cycles of neoadjuvant chemotherapy by tumor reduction rate: a retrospective case-control study. J Breast Cancer. (2023) 26:136. doi: 10.4048/jbc.2023.26.e12, PMID: 37051647 PMC10139844

[15] Liang T Hu B Du H Zhang Y . Predictive value of T2−weighted magnetic resonance imaging for the prognosis of patients with mass−type breast cancer with peritumoral edema. Oncol Lett. (2020) 20:314. doi: 10.3892/ol.2020.12177, PMID: 33133250 PMC7590426

[16] Choi Y Jung K . Normalization of the tumor microenvironment by harnessing vascular and immune modulation to achieve enhanced cancer therapy. Exp Mol Med. (2023) 55:2308–19. doi: 10.1038/s12276-023-01114-w, PMID: 37907742 PMC10689787

[17] Partridge SC Zhang Z Newitt DC Gibbs JE Chenevert TL Rosen MA . Diffusion-weighted MRI findings predict pathologic response in neoadjuvant treatment of breast cancer: the ACRIN 6698 multicenter trial. Radiology. (2018) 289:618–27. doi: 10.1148/radiol.2018180273, PMID: 30179110 PMC6283325

[18] Hottat NA Badr DA Lecomte S Besse-Hammer T Jani JC Cannie MM . Assessment of diffusion-weighted MRI in predicting response to neoadjuvant chemotherapy in breast cancer patients. Sci Rep. (2023) 13:614. doi: 10.1038/s41598-023-27787-x, PMID: 36635514 PMC9837175

[19] Boudreau D Santos F Robidoux A Boileau J-F . Early breast cancer response to neoadjuvant chemotherapy: defining the optimal timing and response rate using clinical tumor measurement. J Clin Oncol. (2015) 33:121. doi: 10.1200/jco.2015.33.28_suppl.121

[20] Chen S Zheng B Tang W Ding S Sui Y Yu X . The longitudinal changes in multiparametric MRI during neoadjuvant chemotherapy can predict treatment response early in patients with HER2-positive breast cancer. Eur J Radiol. (2024) 178:111656. doi: 10.1016/j.ejrad.2024.111656, PMID: 39098252

[21] Bae MS Shin SU Ryu HS Han W Im S-A Park I-A . Pretreatment MR imaging features of triple-negative breast cancer: association with response to neoadjuvant chemotherapy and recurrence-free survival. Radiology. (2016) 281:392–400. doi: 10.1148/radiol.2016152331, PMID: 27195438

[22] Chen Y Wang L Luo R Liu H Zhang Y Wang D . Focal breast edema and breast edema score on T2-weighted images provides valuable biological information for invasive breast cancer. Insights Imaging. (2023) 14:73. doi: 10.1186/s13244-023-01424-7, PMID: 37121926 PMC10149534

[23] Zhao S Li Y Ning N Liang H Wu Y Wu Q . Association of peritumoral region features assessed on breast MRI and prognosis of breast cancer: a systematic review and meta-analysis. Eur Radiol. (2024) 34:6108–20. doi: 10.1007/s00330-024-10612-y, PMID: 38334760

